# Identification of international metastatic renal cell carcinoma database consortium (IMDC) intermediate-risk subgroups in patients with metastatic clear-cell renal cell carcinoma

**DOI:** 10.18632/oncotarget.27762

**Published:** 2020-12-08

**Authors:** Annalisa Guida, Gwénaël Le Teuff, Carolina Alves, Emeline Colomba, Vincenzo Di Nunno, Lisa Derosa, Ronan Flippot, Bernard Escudier, Laurence Albiges

**Affiliations:** ^1^University of Modena and Reggio Emilia, Modena, Italy; ^2^Department of Medical Oncology, Institute Gustave Roussy, Université Paris-Saclay, Villejuif, France; ^3^Service de Biostatistique et d'Épidémiologie, Gustave Roussy, Villejuif, France; ^4^CESP, Faculté de médecinec-Université Paris-Sud, Faculté de médecine - INSERM U1018, Université Paris-Saclay, Villejuif, France; ^5^INSERM U1015, Institute Gustave Roussy, Villejuif, France

**Keywords:** metastatic clear-cell renal cell carcinoma, IDMC, intermediate-risk, heterogeneous prognostic, platelets

## Abstract

Majority of patients with clear-cell renal cell carcinoma (ccRCC) at first line (1L) treatment are classified in the intermediate-risk (IR) subgroup according to International Metastatic Renal Cell Carcinoma Database Consortium (IMDC) score. As these patients have different prognosis, the aim of this study is to better characterize IR patients in order to better tailor the treatment. Retrospective analysis was performed from IGReCC (Institut Gustave Roussy Renal Cell Carcinoma) database. Overall survival (OS) was defined from start of 1L therapy to death or last follow-up. A multivariable Cox model with backward selection procedure (α = 0.01) and a Classification and Regression Tree (CART) analysis were performed to identify which prognostic factors were associated to OS in IR patients.

From 2005 to 2017, 777 patients with ccRCC were treated with an anti-VEGF 1L therapy. Among 571 evaluable patients for IMDC score, 290 (51%) were classified as IR. With median follow-up 5.8 years (min: 0, max: 12.4) 212 deaths (73%) were observed and median OS was 25 months. Only platelet count was significantly associated to OS (hazard ratio 1.88 [95% CI 1.27–2.88] *p* = 0.0017). Median OS for patients with PLT > UNL was 18 months [95% CI 12–23] versus 29 months [95% CI 21.4–35.7] for patients with normal PLT count. The selection of PLT count was confirmed on bootstrap samples and was also selected for the first split of the CART-tree analysis.

Patients in the IR group have a heterogeneous prognosis. Elevated PLT count seems identifies a subgroup of patients with poor outcome in the IMDC intermediate-risk population with ccRCC.

## INTRODUCTION

The risk stratification models for metastatic renal cell carcinoma (mRCC) patients were developed as clinical tool to guide counseling, to predict individual patient prognosis and also to design clinical trial. The International Metastatic renal cell carcinoma Database Consortium (IMDC) score is currently used as prognostic index to stratify patients with mRCC in three subgroups: good, intermediate and poor-risk groups [1, 2]. The model includes six negative clinical prognostic factors: performance status (< 80 Karnofsky Performance Status [KPS]), hemoglobin level < low normal level [LNL]), time from diagnosis to start of systemic treatment [DTT] (< 1 year), corrected serum calcium (> upper normal level [UNL]), neutrophil count (> UNL) and platelet count (> UNL). Patients lacking these negative factors have a good prognosis and may reached a longer survival; patients presenting 1 or 2 factors have an intermediate risk of death with a median overall survival (OS) about 23 months; patients with 3 or more factors have an expected poor risk outcome with median survival about 8 months [2].

During the last decade, anti-VEGF pathway inhibition represented the mainstay front-line treatment of mRCC. Two tyrosine-kinase inhibitors (TKIs), sunitinib and pazopanib, were effective to reduce tumor burden of disease and to prolong progression free survival (PFS), allowing a durable control of metastatic disease in a certain number of patients, regardless of prognostic score stratification. Only in the poor risk group the decision-making algorithm was different: these patients were not candidate for upfront cytoreductive nephrectomy and in selected cases could benefit of mTOR inhibitor temsirolimus in first-line setting [3].

Since 2017, the scenario substantially changed, basically due to two main factors: 1) immunotherapy using check point inhibitor (CPI), alone or in combination, has now integrated all guidelines based on OS benefit both in first and second line setting; 2) treatment strategy and approval of nivolumab plus ipilimumab was delineate by IMDC risk group classification. In particular, two trials focused on intermediate and poor-risk group population. In the phase III trial Checkmate-214 nivolumab plus ipilimumab immunotherapy combination significantly prolonged OS versus sunitinib (median OS not reached versus 28 months, respectively; hazard ratio (HR) 0.63 [99.8% CI, 0.44 to 0.89]; *p* < 0.001) in intermediate and poor-risk untreated patients with mRCC [4]. With a median follow up of 42 months, median OS was 47 months versus 26.6 months in the sunitinib group, (HR 0.66 [95% CI 0.55 to 0.90]; *p* < 0.0001) [5–7]. The CABOSUN phase II randomized trial reported on PFS benefit of cabozantinib over sunitinib (median PFS 8.6 months and 5.3 months, respectively; HR 0.48 [95% CI, 0.31 to 0.74]) in intermediate and poor-risk patients in first-line setting [8].

Furthermore the classification of IMDC may help to define candidate for cytoreductive nephrectomy in upfront metastatic patients [9–11].

Unlike the checkmate 214 trial, 2 studies using a combination of VEGFR-TKI axitinib plus CPI demonstrated benefit over sunitinib in an unselected population in first line. Namely axitinib plus pembrolizumab in the KEYNOTE 426 trial demonstrated better OS (HR 0.53 [95% CI, 0.38 to 0.74]; *p* < 0.0001), PFS (HR 0.69 [95% CI, 0.57 to 0.84]; *p* < 0.0001) and response rate (RR) (59.3% versus 35.7%; *p* < 0.0001) than sunitinib [12]. Axitinib plus avelumab in the JAVELIN RENAL 101 trial reported PFS (median 13.8 versus 8.4 months; HR 0.69 [95% CI 0.56 to 0.84]; *P* < 0.0001) and RR benefit (51.4% versus 25.7%) over sunitinib [13, 14].

Both combinations were FDA approved and, more recently, EMA approved in all IMDC risk group.

The majority of patients with mRCC at first-line treatment are classified in the intermediate-risk group according to IMDC classification accounting for up to 60% of patients in different datasets. IMDC intermediate population encompasses a heterogeneous population of patients with mRCC with a wide spectrum of prognostic outcome, resulting by the presence of one or two laboratory finding or clinical characteristics, or both. The aim of this study was to better characterize intermediate-risk group and to identify a prognostic classification scheme in order to optimize treatment selection.

## RESULTS

### Population characteristics

**Figure 1:**
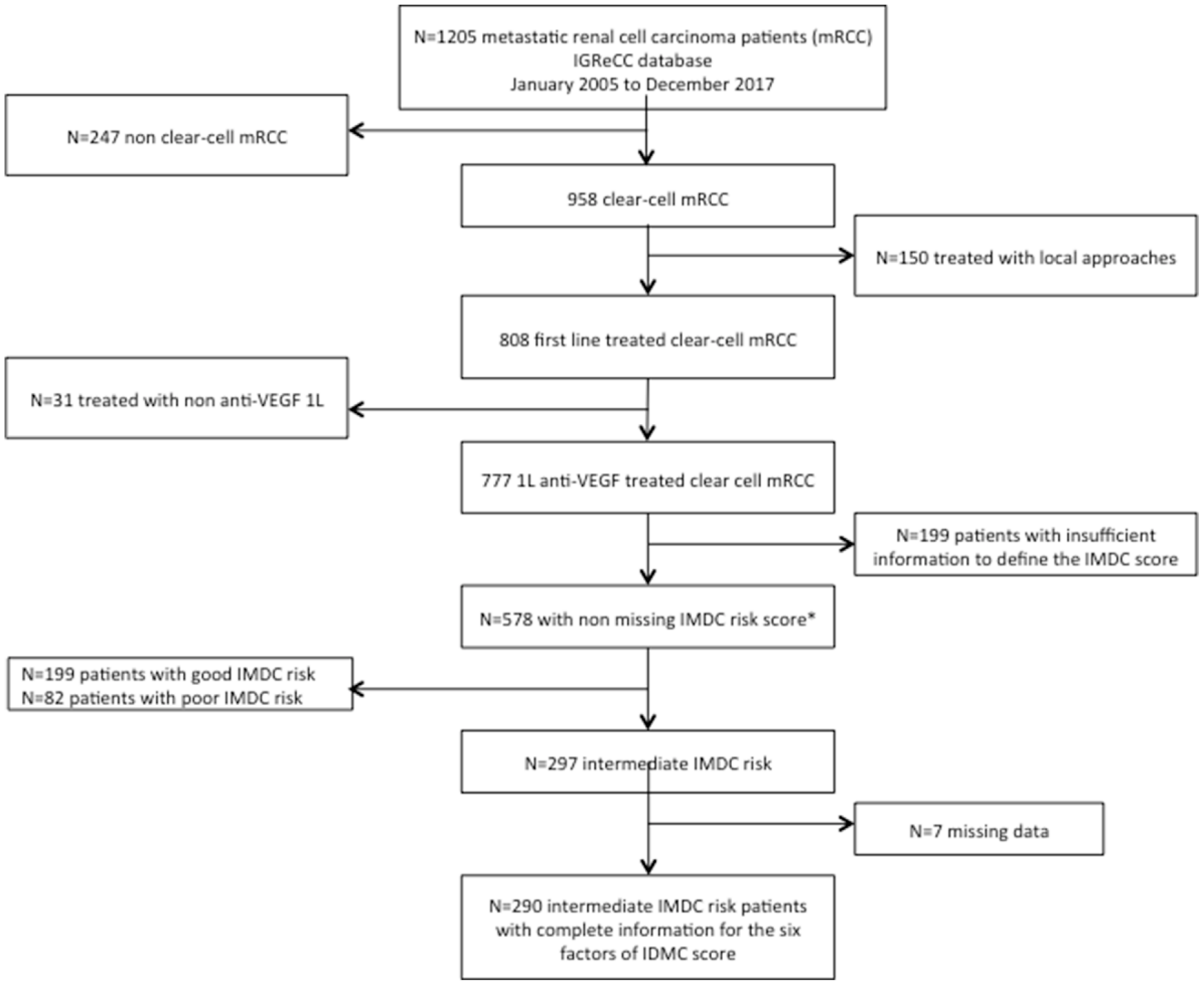
Flowchart for the selection of the study population. IMDC risk score can be assigned to patients even when one of the six prognostic factors is missing since a patient is classified in the intermediate (and poor) IMDC risk score when he has one or two (higher or equal to three) prognostic factors. Abbreviations: IGReCC, Institut Gustave Roussy Renal Cell Carcinoma; mRCC, metastatic renal cell carcinoma; IMDC, International Metastatic Renal Cell Carcinoma Database Consortium.

A data extraction of IGReCC dataset including 1205 patients was performed in December 2017. Clear-cell histology was reported in 958 patients, among these 808 received a front-line systemic treatment. Finally, we retrospectively identified 777 patients with metastatic clear-cell renal cell carcinoma (mccRCC) treated with an anti-VEGF as first-line therapy between January 2005 and December 2017. Of these, 578 patients were evaluable for IMDC score: 199 (34%) patients were classified as good risk, 297 (51%) as intermediate risk and 82 (14%) as poor risk (Figure 1). Among the 290 patients, 130 patients (44.8%) were treated in clinical trials. The median follow-up was 5.8 years (min: 0, max: 12.4). Patients’ characteristics excluded because of missing data to define the IMDC risk score (*n* = 199) were reported in Supplementary Table 1. The baseline characteristics of the population study patients classified in the intermediate IMDC risk score with complete information on the six risk factors (*n* = 290) are reported in Table 1. The median age of population was 58 years old (range: 25–82). All patients have clear-cell histology (100%) with about 13% of them presenting sarcomatoid component. The majority of patients underwent nephrectomy (85%). Patients with one and two prognostic factor were 179 (62%) and 111 (38%), respectively. The most common prognostic factor was DTT < 1 year (69%) while only 13 patients (4%) have KPS < 80%. Hemoglobin level < LNL was the most common factors among laboratory findings. We further studied the correlations between the six prognostic factors used for IMDC score: DTT was associated to other prognostic factors: KPS, hemoglobin level, calcium level, platelet count and neutrophil count (*p* < 0.001). Subsequently, associations between KPS and calcium level (*p* < 0.05) and between hemoglobin and neutrophil count (*p* < 0.001) were identified.

**Table 1:** First-line intermediate-risk mRCC patients characteristics (*n* = 290)

Characteristics	*N* = 290 (%)
Age at diagnosis, median (min–max)	58 (25–82)
Gender	
Male	227 (78)
Female	63 (22)
Prior Nephrectomy	
No	44 (15)
Yes	246 (85)
Furhman grade	
Grade 1–2	66 (26)
Grade 3–4	190 (74)
Missing	34
Sarcomatoid Features	
No	24 (40)
Yes	36 (60)
Missing	230
Bone metastases	
No	213 (74)
Yes	76 (26)
Missing	1
Liver metastases	
No	229 (79)
Yes	60 (21)
Missing	1
Brain metastases	
No	275 (96)
Yes	12 (4)
Missing	3
Number of metastatic sites	
0–1	79 (27)
2	85 (29)
> 2	126 (44)
Synchronous metastases	
No	125 (44)
Yes	162 (56)
Missing	3
**Risk factors**	
Karnofky Performance Status < 80%	13 (4)
Time from diagnosis to treatment < 1 year	200 (69)
Hemoglobin level < LNL	121 (42)
Neutrophils level > UNL	30 (10)
Platelets counts > UNL	29 (10)
Calcium level > UNL	8 (3)
Number of prognostic factors	
1 prognostic factor	179 (62)
2 prognostic factors	111 (38)

Abbreviations: mRCC, metastatic renal cell carcinoma; UNL, upper normal level; LNL, low normal level.

### Prognostic analysis

**Figure 2:**
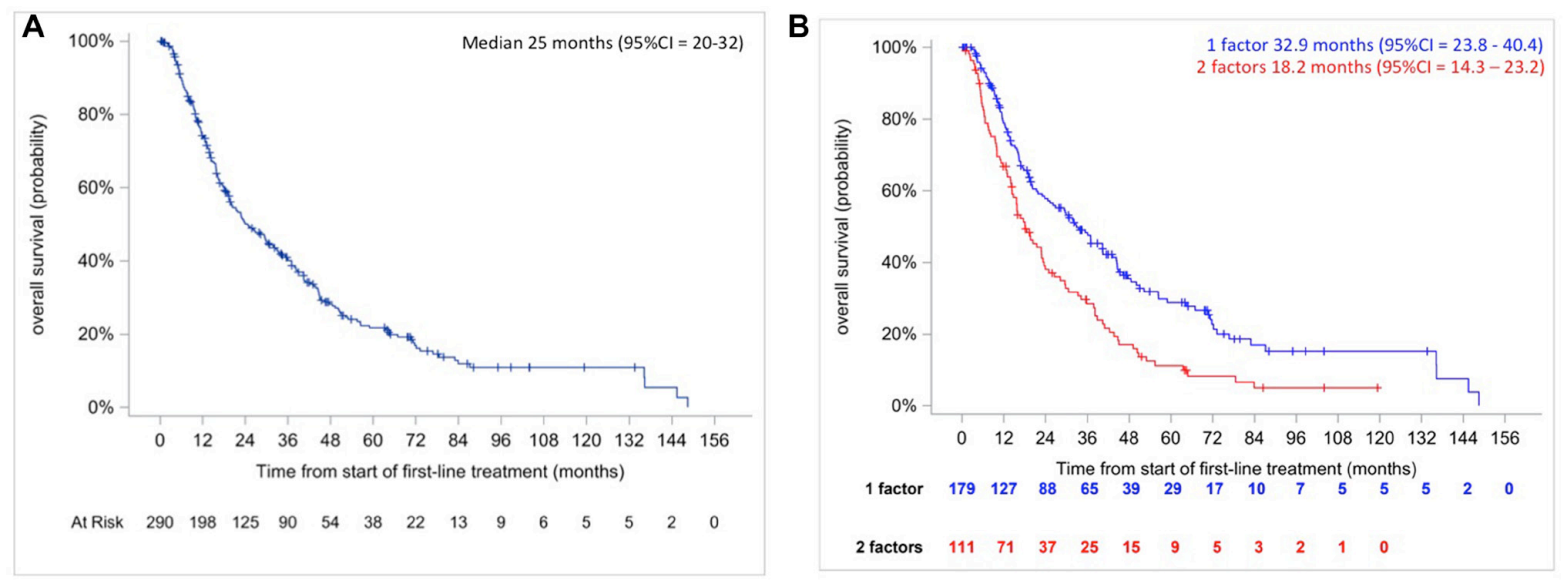
Kaplan–Meier overall survival curves in intermediate IMDC risk patients (**A**, right) and according to the number of prognostic factors (**B**, left).

We observed 212 (73%) deaths and the median OS was 25 months (95% CI, 20 to 32) (Figure 2A).

#### Approach 1: Classification based on the number of risk factors

Median OS was longer for patients with one prognostic factor versus 2 prognostic factors: 33 months (95% CI, 24 to 40) versus 18 months (95% CI, 14 to 23) (Figure 2B, *p* < 0.0001, log-rank test). The hazard ratio was HR_2/1_ = 1.73 [95% CI = 1.314–2.278] *p* < 0.0001 and c-index was 0.5665. There is no violation of the proportional hazards assumption (*p* = 0.6575, Grambsh & Therneau test).

#### Approach 2: Classification based on the multivariable Cox regression model with backward selection

**Figure 3:**
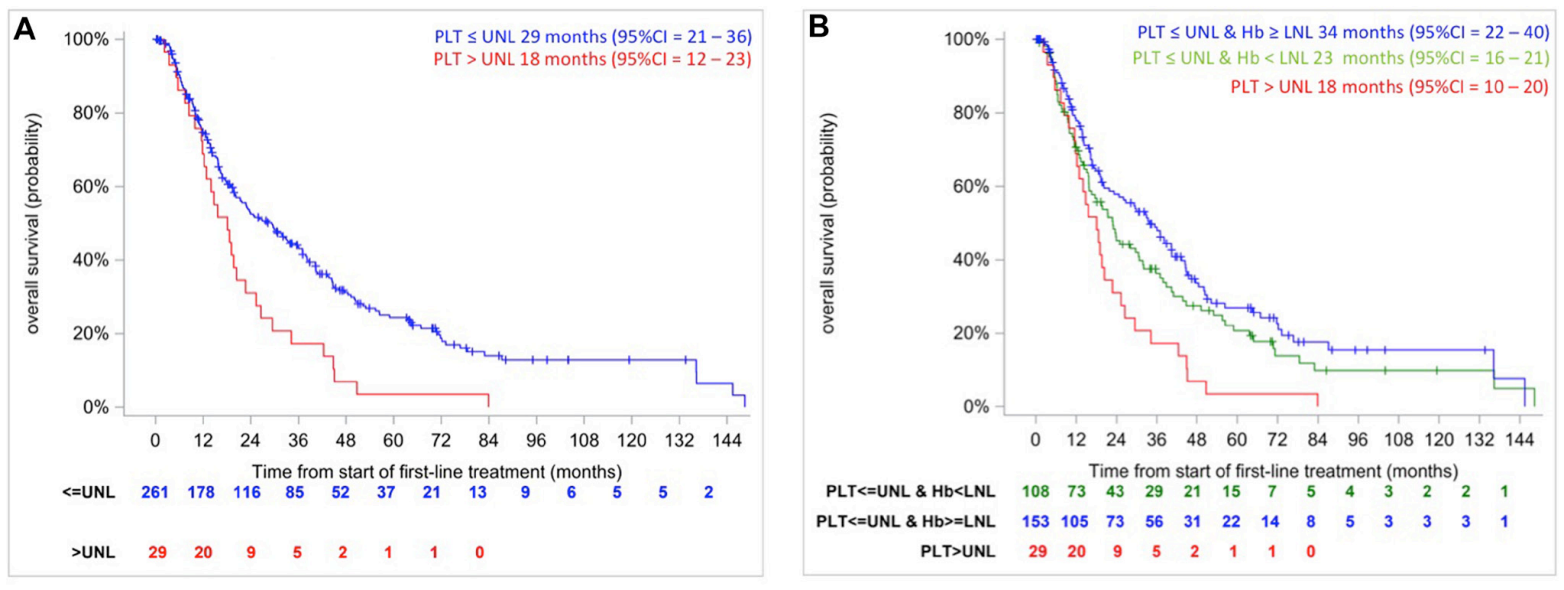
Kaplan–Meier overall survival curves in intermediate IMDC risk patients according to the platelet count (≤ ULN, > ULN) (**A**) and according to resulting from CART algorithm (**B**).

The multivariable Cox model with a backward selection procedure showed that the only prognostic factor associated with OS was platelet count (HR_>ULN vs ≤ULN_ 1.88 [95% CI, 1.27 to 2.79]; *p* = 0.002) with no violation of the proportional hazards assumption (*p* = 0.2235, Grambsh & Therneau test). The c-index was 0.5274. This indicates that the risk of death in patients with platelets counts > ULN (*n* = 29) is higher (median OS: 18 months [95% CI, 12 to 23]) compared as patients with normal platelet count (*n* = 261) (median OS: 29 months [95% CI, 21 to 36]) (Figure 3A, *p* = 0.0014, log rank test). A robustness analysis based on 1000 bootstrap resampling confirms this result with a very high percentage (80% and 96% for *p* = 0.01 and 0.05, respectively) of the number of bootstrapped samplings where platelet was significantly associated to OS. This percentage varied from 5 to 27% for *p* = 0.01 and from 18 to 60% for *p* = 0.05 for the other prognostic factors (Table 2). For *p* = 0.05, DTT and hemoglobin were selected in more than 50% of cases. No interaction allows improving the goodness-of-fit or discriminant ability of the previous model (data not shown).

**Table 2:** Multivariable Cox model including the six predictors of the IMDC score with backward selection procedure: percentage of selection of significant predictors

IMDC factors	Alpha = 0.01 Bootstrap	Alpha = 0.05 Bootstrap
Platelets count	80%	96%
Hemoglobin level	27%	60%
Time from diagnosis to treatment < 1 year	20%	51%
Calcium level	23%	48%
Karnofky Performance Status	15%	31%
Neutrophils count	5%	18%

#### Approach 3: Classification based on the CART method

**Figure 4:**
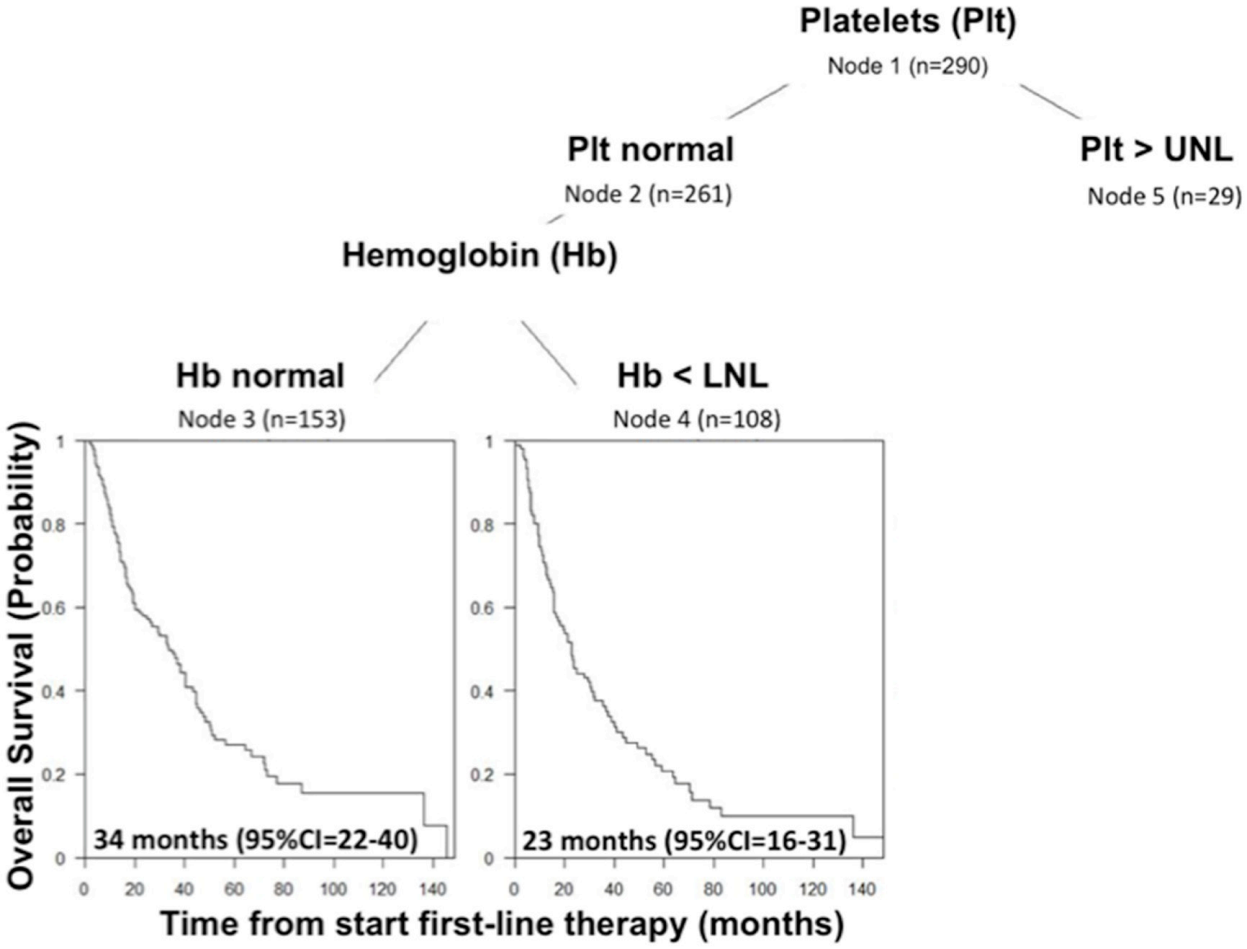
CART-Tree analysis for overall survival in IMDC intermediate risk group.

Figure 4 represents the resulting CART-tree for OS and the first split is based on platelet count (Node 1), which is the most important prognostic factor. This finding was in accordance with the previous analyses. Patients with platelet count ≤ ULN (Node 2) are further split by hemoglobin value that represents the second most relevant risk factor (Node 3 and 4). The fifth node was represented by patients with platelets count > ULN, but further split contained less than 20 patients and was not considered. Through this analysis, CART-tree method clearly identifies a prognostic classification scheme with 3 classes:

• Class 1: patients with platelets counts ≤ ULN and normal hemoglobin (*n* = 153, 99 deaths).

• Class 2: patients with platelets counts ≤ ULN and hemoglobin level < LNL (*n* = 108, 84 deaths)

• Class 3: patients with platelets counts > UNL (*n* = 29, 29 deaths)

Class 1 consists of 153 patients with the best prognosis: median OS was 34 months [95% CI, 22 to 40]. In Class 2, 108 patients were identified with an intermediate prognosis: median OS was 23 months [95% CI, 16 to 31]. Finally, Class 3 represents the smallest groups consisting in 29 patients with the poorest prognosis: median OS was 18 months [95% CI, 10 to 20] (Figure 3B, *p* = 0.0019, log-rank test). The hazards ratios were HR_class 2 vs class 1_ = 1.27 [95% CI, 0.95–1.70] and HR_class 3 vs class 1_ = 2.08 [95% CI, 1.37–3.16] and the c-index was 0.55 (no violation of the proportional hazards assumption, *p* = 0.4506 and 0.3701, Grambsh & Therneau test). However, considering the result of the robustness analysis of the approach 2, the second split using hemoglobin may be uncertain.

### Comparison of the three approaches

The prognostic classification schemes developed by the three approaches were similar in terms of goodness-of-fit (AIC = 2019.0, 2025.3 and 2024.9 for approaches 1, 2 and 3, respectively) although the lowest AIC was provided by the approach 1 counting the number of prognostic factors. In terms of the discriminant ability, the approaches 1 and 3 have similar c-index (0.57 versus 0.55, respectively) compared to 0.53 for approach 2.

## DISCUSSION

The CPI based regimen has transformed the first-line treatment approach in mRCC management, with two possible distinct approved approaches: doublet of CPI in intermediate and poor IMDC risk patients or VEGF/TKI plus CPI in mRCC patients regardless of IMDC risk group [4, 12, 13]. The opportunity of these two distinct approaches raise the question of patient selection and therefore which combination should be offered to which patient [15, 16]. Our study focused on prognostic features of patients in IMDC-based intermediate-risk group, which represents the most frequent risk population.

In the past years both MSKCC and IMDC scores were used almost exclusively to define prognosis of patients with mRCC, with the only exception of poor-risk group patients susceptible of treatment with temsirolimus [17].

Originally, MSKCC classification was created for patients treated with cytokine therapy and later it was also validated for the VEGF-target therapy. IMDC score was proposed in 2009 for prognostic definition of patients with mRCC treated with first line VEGF-target therapy. Subsequently, it was also validated in further lines of treatment. Several attempts to improve prognostic power of these scores, with the addition of more factors, were proposed but IMDC in its initial design remains the most important prognostic tools in mRCC.

In the most recent immune checkpoint inhibitors era both MSKCC and IMDC classification were not overcome, on the contrary, their prognostic role was confirmed again and moreover a potential predictive role has emerged [4, 8, 10]. First of all, they were used to conceive and design recent clinical trials (CABOSUN, Checkmate-214, and CARMENA) with an evolving predictive use of these scores. Currently, cabozantinib or nivolumab plus ipilimumab combination may be offered as first line treatment in a patient with mRCC stratified as intermediate-risk profile. Prognostic classification may also define front line surgical strategy, because patients defined as poor-risk were not candidate to cytoreductive nephrectomy as front-line. In Chekmate 214 trial both MSKCC and IMDC were used to define patients prognosis with reproducible predictive power of the model even in the CPI era.

Intermediate-risk class requires additional prognosis tool to further segregate this heterogeneous group into sub-groups in order to better tailor the treatment. In our cohort, overall survival was 25 months (95% CI, 20 to 32), in line with recent sunitinib control arms from Checkmate 214 and CABOSUN trial [4, 8]. We reported that one versus two prognostic factors is significantly associated with a better outcome: 33 months (95% CI, 24 to 40) versus 18 months (95% CI, 14 to 23). So far, few similar experiences exploring intermediate-risk group of mRCC were conducted. Sella et al. reported a retrospective analysis from 6 randomized clinical trials, showing that number of prognostic factors (one versus two) and ECOG PS significantly affect survival within this subgroup population. Indeed, the authors described 517 IMDC classified mRCC patients treated in first line and second line, and reported median OS of 27.8 (95% CI, 24 to 30.2) versus 15 months (95% CI, 12.9 to 16.7) in patients with 1 versus 2 risk factors (HR 0.51 [95% CI, 0.41 to 0.64] [18].

Iacovelli et al. analyzed both intermediate and poor-risk populations (457 patients) in first-line setting founding that the presence of one versus two prognostic factors was significantly associated with overall survival and seems to stratify intermediate-risk group: 32.8 months (95% CI, 28.9 to 36.9) versus 20 months (95% CI, 15.7 to 24.4) [19]. Tamada et al. explored potential prognostic factors within intermediate-risk group reporting similar results as well. Median OS was significantly longer in patients with one prognostic factor versus two prognostic factors (43.6 versus 22.5; HR 1.93 [95% CI 1.12–3.33]; *p* = 0.017 [20].

In our study, platelet count was the more important prognostic factor, able to stratify intermediate-risk population in two subgroups with median overall survival 29 months versus 18 months (HR 1.88 [95% CI, 1.27 to 2.79]; *p* = 0.002), respectively. In addition we performed CART analysis, a technique more suitable to generation of clinical decision rules. It confirms the relevant prognostic role of platelet count and provide the classification of three different prognostic subgroups: patients with normal platelet counts and hemoglobin, patients with normal platelet counts and hemoglobin level < LNL and patients with platelets counts > UNL (median OS, respectively, 34 months [95% CI, 22 to 40], 23 months [95% CI, 16 to 31] and 18 months [95% CI, 10 to 20]).

Kin et al. retrospectively analyzed intermediate and poor-risk populations focusing on prognostic role of timing of metastatic disease. They founded that metachronous versus synchronous metastases appearance is associated with a better outcome [21]. Finally, data by Takamastu et al. showed that C-reactive protein (PCR) might divide intermediate group in two prognostic subgroups where high level of PCR is associated with a poor outcome [22]. The most explored parameter was the number of prognostic factors, which unfortunately is redundant in the construction algorithm of prognostic models. Indeed, IMDC score is already the result from the sum of number of prognostic factors.

Ultimately, it is anticipated that distinct biologically and molecularly defined population drives the heterogeneity of this intermediate population. de Velasco et al. demonstrated that a 34-gene signature model improved the prognostic predictive power of the IMDC model in patients with metastatic clear cell RCC [23]. More recently, the IMmotion 150 and IMmotion 151 translational programs distinguished angiogenesis and Teff driven signatures that may capture the distinct underlying main drivers that the IMDC risk group classification may not capture [24–26]. In a rapidly evolving field where both approach are approved in first line: doublet of immune checkpoint versus combination of VEGFR-TKI with an immune checkpoint, the question of treatment selection is debated and new biologic rationales are emerging [27]. While distinct biology may contribute to some extent to distinct IMDC disease, our ability to segregate intermediate population has the impact to guide our treatment selection toward on or the other of these 2 approaches.

Our retrospective analysis has several limitations, among which the unicentric database which limits the generalizability of our findings, however we choose to focus on the specific question of first line patient population to address the new clinically relevant question and we selected clear-cell histology and anti-VEGF drug therapies to reduce the possible differences in histology or treatments. More important to note, we performed a robustness statistical analysis with multivariable Cox model with a backward selection procedure, bootstrapping samples and CART analysis. In addition, we kept both alpha error 0.05 and the lower 0.01 with the aim to report stronger evidences. Other limitation of our analysis is represented by the small number of patients with higher platelet count (*N* = 29). This represents a percentage of 10% in line with other publications. Previous CART analysis were constructed with a cut-off of *N* = 20 patients [28, 29]. Our findings are consistent with biological and clinical evidences supporting that platelet count may reflects the systemic inflammatory status and may provide valuable insight in and CPI based treatment era. Prognostic role of thrombocytosis, as confirmed by our results, is well established in several cancer types and it was described in 10–57% of oncologic patients [30]. In 2015, Gu et al. reported data from a systematic review and meta-analysis evaluating the association of elevated platelet count with patients survival in mRCC. The authors concludes that in metastatic clear-cell setting thrombocytosis was associated with poor overall survival (HR 2.05 [95% CI 1.04–4.03] *p* = 0.038) [31].

It is known that tumor cells secrete a widely spectrum of substance, including interleukin-1 (IL-1), interleukin-6 (IL-6), granulocyte-macrophage colony-stimulating factor (GM-CSF), granulocyte colony-stimulating factor (G-CSF), able to mediate the interaction with platelet activation. Not only, it was clarified that platelets may contribute to cancer progression through different mechanisms. Platelets have a crucial role in vascular invasion by releasing of proteolytic enzymes and by activating of platelet aggregation. It was demonstrated that tumor-induced platelet aggregation provides protection and survival advantage to tumor cells by covering cancer cell from physical defenses and immune system [32]. In addition, it is well known that tumor-platelet aggregates are able to embolize and disseminate in the vessels. The role of platelets in angiogenesis is significant, because they represent one of the most important sources of vascular-endothelial growth factor (VEGF). Indeed, during normal physiologic conditions platelets promote angiogenesis to heal wounds, with concurrent activation of inhibition processes. However, the exact role of platelets in tumor-associated angiogenesis still needs to be investigated and clarified [33].

Moreover, in both physiologic and cancer conditions, platelets interact with many cellular types to mediate inflammation and immune response, although platelet interaction with anti cancer T cell immunity is not well known yet. So far, several mechanisms are postulated and more other needs to be described. Platelets seem to contain high levels of TGFβ (transforming growth factor receptor β), a multipotent cytokine, mainly involved in chemotaxis and able to promote differentiation of regulatory T cell (T_reg_). According to latest data, TGFβ appears to be strongly related to platelets activation. Furthermore, it was recently demonstrated that platelets GARP-TGFβ complex (glicoprotein A repetitions predominant-TGF β) play a negative role in antitumor T cell immunity [34]. In addition, platelets seem to release in microenvironment soluble factors able to suppress the activation and effector function of T cell, particularly blocking CD8^+^ T cell proliferation and interferon-γ production.

Interestingly, it was described that platelet count may play both a prognostic and predictive role in CPI era. Elevated pre-treatment platelet/lymphocytes ratio (PLR) is associated with shorter OS, shorter progression-free survival and with lower response rates in patients with metastatic non-small cell lung cancer and mRCC treated with nivolumab in second or more line of therapy [35, 36].

Given the rapidly evolving field of systemic treatment in mRCC, one of the most important challenges in mRCC is how prognostic stratification will guide front-line treatment selection. Additionally characterization of heterogeneous IMDC intermediate-risk group of patients should be seeked for optimal clinical trials design and stratification. High platelet count reflecting the cancer-related inflammatory status and seems to segregate patients with worst prognosis in the intermediate-risk group. Further analyses are ongoing to validate these findings in patients receiving first line CPI based combination in first line.

## MATERIALS AND METHODS

### Population

IGReCC (Institut Gustave Roussy Renal Cell Carcinoma) database is a single institution collection of data of patients with mRCC. From January 2005 to December 2017, all consecutive patients with mRCC treated at Gustave Roussy were included in the database. Study data were collected and managed using REDCap electronic data capture tools hosted at Gustave Roussy [37].

In this population-based analysis we retrospectively identified patients with metastatic clear cell renal cell carcinoma (mccRCC) who received a first-line therapy with an anti-vascular endothelial growth factor (anti-VEGF)/anti VEGF receptor drug.

We collected demographic, baseline patient characteristics and overall survival data at first-line with anti-VEGF therapy. Patients with unavailable baseline IMDC score stratification were excluded from the analysis. Only patients with intermediate IMDC score were included in the final analysis.

### Statistical analysis

We described the patients’ characteristics (gender, age at diagnosis, Karnofsky Performance Scale (KPS), prior nephrectomy, Furhman grade, sarcomatoid features, synchronous metastases, number of metastastic sites, presence of bone metastases) in the IMDC-based intermediate risk group. OS is defined from the start of first-line treatment to death; patients alive were censored at the date of last follow-up. To identify prognostic classification schemes with different prognoses (in terms of OS) within this intermediate IMDC risk group, we used three statistical approaches and complete cases patients, i.e., with no missing value for the six IDMC factors. The first approach consists to define a classification scheme from patients with one or two prognostic factors from the six factors defining the IMDC score. The second approach is a multivariable Cox model with a backward selection procedure including the six prognostic factors that defined the IMDC score. We completed it by performing a robustness analysis: it consists to repeat the same analysis applying the backward Cox model in bootstrapping samples and to compute the percentage that a prognostic factor is significantly associated to OS among these bootstrapping samples. The cut-offs 0.05 and 0.01 will be used for the selection. The third approach is the classification and regression tree (CART) [28, 29, 38, 39]. The principle of CART algorithm is to partition the population into homogeneous sub-populations, recursively. In the first step, the population is splitted into two parts according to a prognostic factor that shows the largest difference in prognosis, tested using the log-rank test. This procedure is repeated for the resulting two subpopulations. As stop criterion we used a 5% significance level and no further split was allowed for a node containing less than 20 patients. Such subpopulations are called ‘final nodes’ [28, 29, 38].

The prognostic value of the different classifications was illustrated by representing OS estimated by the Kaplan–Meier technique and median with its 95% confidence interval (CI). The statistical analyses were performed with the SAS software 9.4 (SAS Institute) and rpart R package for the CART analysis [40].

## SUPPLEMENTARY MATERIALS



## References

[1] Heng DY , Xie W , Regan MM , Warren MA , Golshayan AR , Sahi C , Eigl BJ , Ruether JD , Cheng T , North S , Venner P , Knox JJ , Chi KN , et al. Prognostic factors for overall survival in patients with metastatic renal cell carcinoma treated with vascular endothelial growth factor-targeted agents: results from a large, multicenter study. J Clin Oncol. 2009; 27:5794–9. 10.1200/JCO.2008.21.4809. 19826129

[2] Heng DY , Xie W , Regan MM , Harshman LC , Bjarnason GA , Vaishampayan UN , Mackenzie M , Wood L , Donskov F , Tan MH , Rha SY , Agarwal N , Kollmannsberger C , et al. External validation and comparison with other models of the International Metastatic Renal-Cell Carcinoma Database Consortium prognostic model: a population-based study. Lancet Oncol. 2013; 14:141–8. 10.1016/S1470-2045(12)70559-4. 23312463 PMC4144042

[3] Escudier B , Porta C , Schmidinger M , Rioux-Leclercq N , Bex A , Khoo V , Gruenvald V , Horwich A , Committee EG , and ESMO Guidelines Committee. Renal cell carcinoma: ESMO Clinical Practice Guidelines for diagnosis, treatment and follow-up. Ann Oncol. 2016; 27:v58–v68. 10.1093/annonc/mdw328. 27664262

[4] Motzer RJ , Tannir NM , McDermott DF , Aren Frontera O , Melichar B , Choueiri TK , Plimack ER , Barthelemy P , Porta C , George S , Powles T , Donskov F , Neiman V , et al, and CheckMate 214 Investigators. Nivolumab plus Ipilimumab versus Sunitinib in Advanced Renal-Cell Carcinoma. N Engl J Med. 2018; 378:1277–90. 10.1056/NEJMoa1712126. 29562145 PMC5972549

[5] Tannir NM , Arén Frontera O , Hammers HJ , Carducci MA , McDermott DF , Salman P , Escudier B , Beuselinck B , Amin A , Porta C , George S , Bracarda S , Tykodi SS , et al Thirty-month follow-up of the phase III CheckMate 214 trial of first-line nivolumab + ipilimumab (N+I) or sunitinib (S) in patients (pts) with advanced renal cell carcinoma (aRCC). J Clin Oncol. 2019; 37:547 10.1200/JCO.2019.37.7_suppl.547. 30650044

[6] Tannier NM , McDermott DF , Escudier B , Hammers HJ , Aren OR , Plimack ER , Barthelemy P , Neiman V , George S , Porta C , Powles T , Donskov F , Grimm MO , et al Overall survival and independent review of response in CheckMate 214 with 42-month follow-up: First-line nivolumab + ipilimumab (N+I) versus sunitinib (S) in patients (pts) with advanced renal cell carcinoma (aRCC). J Clin Oncol. 2020; 38:609 10.1200/JCO.2020.38.6_suppl.609.

[7] Motzer RJ , Rini BI , McDermott DF , Aren Frontera O , Hammers HJ , Carducci MA , Salman P , Escudier B , Beuselinck B , Amin A , Porta C , George S , Neiman V , et al, and CheckMate 214 investigators. Nivolumab plus ipilimumab versus sunitinib in first-line treatment for advanced renal cell carcinoma: extended follow-up of efficacy and safety results from a randomised, controlled, phase 3 trial. Lancet Oncol. 2019; 20:1370–85. 10.1016/S1470-2045(19)30413-9. 31427204 PMC7497870

[8] Choueiri TK , Halabi S , Sanford BL , Hahn O , Michaelson MD , Walsh MK , Feldman DR , Olencki T , Picus J , Small EJ , Dakhil S , George DJ , Morris MJ . Cabozantinib Versus Sunitinib As Initial Targeted Therapy for Patients With Metastatic Renal Cell Carcinoma of Poor or Intermediate Risk: The Alliance A031203 CABOSUN Trial. J Clin Oncol. 2017; 35:591–7. 10.1200/JCO.2016.70.7398. 28199818 PMC5455807

[9] Larcher A , Wallis CJD , Bex A , Blute ML , Ficarra V , Mejean A , Karam JA , Van Poppel H , Pal SK . Individualised Indications for Cytoreductive Nephrectomy: Which Criteria Define the Optimal Candidates? Eur Urol Oncol. 2019; 2:365–78. 10.1016/j.euo.2019.04.007. 31109902

[10] Mejean A , Ravaud A , Thezenas S , Colas S , Beauval JB , Bensalah K , Geoffrois L , Thiery-Vuillemin A , Cormier L , Lang H , Guy L , Gravis G , Rolland F , et al. Sunitinib Alone or after Nephrectomy in Metastatic Renal-Cell Carcinoma. N Engl J Med. 2018; 379:417–27. 10.1056/NEJMoa1803675. 29860937

[11] Mejean A , Thezenas S , Chevreau C , Bensalah K , Geoffrois L , Thiery-Vuillemin A , Cormier L , Lang H , Guy L , Gravis G , Rolland F , Linassier C , Timsit M , et al Cytoreductive nephrectomy (CN) in metastatic renal cancer (mRCC): Update on Carmena trial with focus on intermediate IMDC-risk population. J Clin Oncol. 2019; 37:4508 10.1200/JCO.2019.37.15_suppl.4508.

[12] Rini BI , Plimack ER , Stus V , Gafanov R , Hawkins R , Nosov D , Pouliot F , Alekseev B , Soulieres D , Melichar B , Vynnychenko I , Kryzhanivska A , Bondarenko I , et al, and KEYNOTE-426 Investigators. Pembrolizumab plus Axitinib versus Sunitinib for Advanced Renal-Cell Carcinoma. N Engl J Med. 2019; 380:1116–27. 10.1056/NEJMoa1816714. 30779529

[13] Motzer RJ , Penkov K , Haanen J , Rini B , Albiges L , Campbell MT , Venugopal B , Kollmannsberger C , Negrier S , Uemura M , Lee JL , Vasiliev A , Miller WH Jr , et al. Avelumab plus Axitinib versus Sunitinib for Advanced Renal-Cell Carcinoma. N Engl J Med. 2019; 380:1103–15. 10.1056/NEJMoa1816047. 30779531 PMC6716603

[14] Choueiri TK , Motzer RJ , Rini BI , Haanen J , Campbell MT , Venugopal B , Kollmannsberger C , Gravis-Mescam G , Uemura M , Lee JL , Grimm MO , Gurney H , Schmidinger M , et al. Updated efficacy results from the JAVELIN Renal 101 trial: first-line avelumab plus axitinib versus sunitinib in patients with advanced renal cell carcinoma. Ann Oncol. 2020; 31:1030–1039. 10.1016/j.annonc.2020.04.010. 32339648 PMC8436592

[15] Albiges L , Powles T , Staehler M , Bensalah K , Giles RH , Hora M , Kuczyk MA , Lam TB , Ljungberg B , Marconi L , Merseburger AS , Volpe A , Abu-Ghanem Y , et al. Updated European Association of Urology Guidelines on Renal Cell Carcinoma: Immune Checkpoint Inhibition Is the New Backbone in First-line Treatment of Metastatic Clear-cell Renal Cell Carcinoma. Eur Urol. 2019; 76:151–6. 10.1016/j.eururo.2019.05.022. 31151678

[16] de Velasco G , Bex A , Albiges L , Powles T , Rini BI , Motzer RJ , Heng DYC , Escudier B . Sequencing and Combination of Systemic Therapy in Metastatic Renal Cell Carcinoma. Eur Urol Oncol. 2019; 2:505–14. 10.1016/j.euo.2019.06.022. 31377308

[17] Hudes G , Carducci M , Tomczak P , Dutcher J , Figlin R , Kapoor A , Staroslawska E , Sosman J , McDermott D , Bodrogi I , Kovacevic Z , Lesovoy V , Schmidt-Wolf IG , et al, and Global ARCC Trial. Temsirolimus, interferon alfa, or both for advanced renal-cell carcinoma. N Engl J Med. 2007; 356:2271–81. 10.1056/NEJMoa066838. 17538086

[18] Sella A , Michaelson MD , Matczak E , Simantov R , Lin X , Figlin RA . Heterogeneity of Patients With Intermediate-Prognosis Metastatic Renal Cell Carcinoma Treated With Sunitinib. Clin Genitourin Cancer. 2017; 15:291–9.e1. 10.1016/j.clgc.2016.08.013. 27638198

[19] Iacovelli R , De Giorgi U , Galli L , Zucali P , Nole F , Sabbatini R , Fraccon AP , Basso U , Mosca A , Atzori F , Santini D , Facchini G , Fornarini G , et al. Is It Possible to Improve Prognostic Classification in Patients Affected by Metastatic Renal Cell Carcinoma With an Intermediate or Poor Prognosis? Clin Genitourin Cancer. 2018; 16:355–9.e1. 10.1016/j.clgc.2018.04.007. 29803346

[20] Tamada S , Iguchi T , Yasuda S , Kato M , Yamasaki T , Nakatani T . The difference in the survival rate of patients with metastatic renal cell carcinoma in the intermediate-risk group of the Memorial Sloan Kettering Cancer Center criteria. Oncotarget. 2018; 9:27752–9. 10.18632/oncotarget.25554. 29963234 PMC6021254

[21] Kim SH , Suh YS , Lee DE , Park B , Joo J , Joung JY , Seo HK , Lee KH , Chung J . A retrospective comparative study of progression-free survival and overall survival between metachronous and synchronous metastatic renal cell carcinoma in intermediate- or poor-risk patients treated with VEGF-targeted therapy. Oncotarget. 2017; 8:93633–43. 10.18632/oncotarget.20674. 29212178 PMC5706824

[22] Takamatsu K , Mizuno R , Omura M , Morita S , Matsumoto K , Shinoda K , Kosaka T , Takeda T , Shinojima T , Kikuchi E , Asanuma H , Oyama M , Mikami S , Oya M . Prognostic Value of Baseline Serum C-Reactive Protein Level in Intermediate-Risk Group Patients With Metastatic Renal-Cell Carcinoma Treated by First-Line Vascular Endothelial Growth Factor-Targeted Therapy. Clin Genitourin Cancer. 2018; 16:e927–e33. 10.1016/j.clgc.2018.03.012. 29678472

[23] de Velasco G , Culhane AC , Fay AP , Hakimi AA , Voss MH , Tannir NM , Tamboli P , Appleman LJ , Bellmunt J , Kimryn Rathmell W , Albiges L , Hsieh JJ , Heng DY , et al. Molecular Subtypes Improve Prognostic Value of International Metastatic Renal Cell Carcinoma Database Consortium Prognostic Model. Oncologist. 2017; 22:286–92. 10.1634/theoncologist.2016-0078. 28220024 PMC5344647

[24] Rini BI , Powles T , Atkins MB , Escudier B , McDermott DF , Suarez C , Bracarda S , Stadler WM , Donskov F , Lee JL , Hawkins R , Ravaud A , Alekseev B , et al, and IMmotion151 Study Group. Atezolizumab plus bevacizumab versus sunitinib in patients with previously untreated metastatic renal cell carcinoma (IMmotion151): a multicentre, open-label, phase 3, randomised controlled trial. Lancet. 2019; 393:2404–15. 10.1016/S0140-6736(19)30723-8. 31079938

[25] Rini BI , Huseni M , Atkins MB , McDermott DF , Powles TB , Escudier B , Banchereau R , Liu LF , Leng N , Fan J , Doss J , Nalle S , Carroll S , et al Molecular correlates differentiate response to atezolizumab (atezo) + bevacizumab (bev) vs sunitinib (sun): Results from a phase III study (IMmotion151) in untreated metastatic renal cell carcinoma (mRCC). Ann Oncol. 2018; 29:viii724–25. 10.1093/annonc/mdy424.037.

[26] McDermott DF , Huseni MA , Atkins MB , Motzer RJ , Rini BI , Escudier B , Fong L , Joseph RW , Pal SK , Reeves JA , Sznol M , Hainsworth J , Rathmell WK , et al. Clinical activity and molecular correlates of response to atezolizumab alone or in combination with bevacizumab versus sunitinib in renal cell carcinoma. Nat Med. 2018; 24:749–57. 10.1038/s41591-018-0053-3. 29867230 PMC6721896

[27] Hirsch L , Flippot R , Escudier B , Albiges L . Immunomodulatory Roles of VEGF Pathway Inhibitors in Renal Cell Carcinoma. Drugs. 2020; 80:1169–1181. 10.1007/s40265-020-01327-7. 32601914

[28] Atkinson E , Therneau T . An introduction to Recursive Partitioning Using the RPART Routines. Mayo Clinic. 2000.

[29] Therneau T , Atkinson E . An introduction to Recursive Partitioning Using the RPART Routines. Mayo Clinic. 1997.

[30] Sierko E , Wojtukiewicz MZ . Platelets and angiogenesis in malignancy. Semin Thromb Hemost. 2004; 30:95–108. 10.1055/s-2004-822974. 15034801

[31] Gu L , Li H , Gao Y , Ma X , Chen L , Li X , Zhang Y , Fan Y , Zhang X . The association of platelet count with clinicopathological significance and prognosis in renal cell carcinoma: a systematic review and meta-analysis. PLoS One. 2015; 10:e0125538. 10.1371/journal.pone.0125538. 25955026 PMC4425534

[32] Bambace NM , Holmes CE . The platelet contribution to cancer progression. J Thromb Haemost. 2011; 9:237–49. 10.1111/j.1538-7836.2010.04131.x. 21040448

[33] Peterson JE , Zurakowski D , Italiano JE Jr , Michel LV , Fox L , Klement GL , Folkman J . Normal ranges of angiogenesis regulatory proteins in human platelets. Am J Hematol. 2010; 85:487–93. 10.1002/ajh.21732. 20575035

[34] Rachidi S , Metelli A , Riesenberg B , Wu BX , Nelson MH , Wallace C , Paulos CM , Rubinstein MP , Garrett-Mayer E , Hennig M , Bearden DW , Yang Y , Liu B , Li Z . Platelets subvert T cell immunity against cancer via GARP-TGFbeta axis. Sci Immunol. 2017; 2:eaai7911. 10.1126/sciimmunol.aai7911. 28763790 PMC5539882

[35] Diem S , Schmid S , Krapf M , Flatz L , Born D , Jochum W , Templeton AJ , Fruh M . Neutrophil-to-Lymphocyte ratio (NLR) and Platelet-to-Lymphocyte ratio (PLR) as prognostic markers in patients with non-small cell lung cancer (NSCLC) treated with nivolumab. Lung Cancer. 2017; 111:176–81. 10.1016/j.lungcan.2017.07.024. 28838390

[36] Retz M , Bedke J , Herrmann E , Grimm MO , Zimmermann U , Müller L , Leiber C , Teber D , Wirth M , Bolenz C , van Alphen R , De Santis M , Beeker A , et al Phase III randomized, sequential, open-label study to evaluate the efficacy and safety of sorafenib-pazopanib versus pazopanib-sorafenib in the treatment of metastatic renal cell carcinoma (SWITCH-II). Ann Oncol. 2017; 28:v295–v329. 10.1093/annonc/mdx371. 30529901

[37] Harris PA , Taylor R , Thielke R , Payne J , Gonzalez N , Conde JG . Research electronic data capture (REDCap)--a metadata-driven methodology and workflow process for providing translational research informatics support. J Biomed Inform. 2009; 42:377–81. 10.1016/j.jbi.2008.08.010. 18929686 PMC2700030

[38] Breiman L , Friedman J , Charles JS , Olshen RA . Classification Regression Trees. New York 1984.

[39] Ciampi A , Lawless JF , McKinney SM , Singhal K . Regression and recursive partition strategies in the analysis of medical survival data. J Clin Epidemiol. 1988; 41:737–48. 10.1016/0895-4356(88)90160-6. 3418363

[40] R Core Team (2018). R: A language and environment for statistical computing. R Foundation for Statistical Computing, Vienna, Austria Available online at https://www.R-project.org/.

