# Supplementary material for: Identification of international metastatic renal cell carcinoma database consortium (IMDC) intermediate-risk subgroups in patients with metastatic clear-cell renal cell carcinoma
Source: Oncotarget. 2020 Dec 8;11(49):4582–92. doi: 10.18632/oncotarget.27762 (PMC7733622; doi:10.18632/oncotarget.27762)
Supplement: Supplementary file 1 [file oncotarget-11-4582-s001.pdf]

Identification of international metastatic renal cell carcinoma database consortium (IMDC) intermediate-risk subgroups in patients with metastatic clear-cell renal cell carcinoma

SUPPLEMENTARY MATERIALS

Supplementary Table 1: Comparison of the characteristics of patients without and with missing values of IMDC risk score

Characteristics	Patient with no missing value of IMDC *N* = 578 (%)	Patients with missing value of IMDC *N* = 199 (%)	*p* value
Age median at diagnosis (min–max)	57.2 (25.2–84.3)	56.6 (27.5–84.1)	0.7703
Gender			
Male	433 (75)	156 (78)	0.3230
Female	145 (25)	43 (22)	
Prior Nephrectomy			
No	73 (13)	9 (5)	0.0013
Yes	505 (87)	190 (95)	
Furhman grade			
Grade 1–2	150 (30)	62 (34)	0.2835
Grade 3–4	348 (70)	118 (66)	
Unknown	80	19	
Sarcomatoid Features			
No	36 (36)	15 (50)	0.1568
Yes	65 (64)	15 (50)	
Unknown	477	169	
Bone metatases			
No	443 (77)	159 (85)	0.0165
Yes	134 (23)	28 (15)	
Unknown	1	12	
Liver metatases			
No	458 (80)	169 (90)	0.0007
Yes	118 (20)	18 (10)	
Unknown	2	12	
Brain metatases			
No	558 (97)	181 (97)	0.8494
Yes	17 (3)	5 (3)	
Unknown	3	13	
Number of metastatic sites			
0–1	135 (23)	86 (46)	< 0.0001
2	208 (36)	56 (30)	
> 2	235 (41)	46 (24)	
Unknown	0	11	

Synchronous metastases			
No	313 (55)	112 (57)	0.5404
Yes	257 (45)	83 (43)	
Unknown	8	4	
**Prognostic factors**			
Karnofky Performance Status			
100–80	538 (94)	47 (100)	0.0769
< 80%	36 (6)	0	
Unknown	4	152	
Time from diagnosis to treatment			
≥ 1 year	306 (53)	99 (50)	0.4368
< 1 year	272 (47)	100 (50)	
Hemoglobin level			
Normal	378 (65)	17 (71)	0.5828
< LNL	200 (35)	7 (29)	
Unknown	0	175	
Neutrophils level			
Normal	511 (88)	11 (92)	0.7264
> UNL	67 (12)	1 (8)	
Unknown	0	187	
Platelets counts			0.8197
Normal	494 (85)	14 (87)	
> UNL	85 (15)	2 (13)	
Unknown	0	183	
Calcium level			
Normal	540 (95)	8 (89)	0.4394
> UNL	30 (5)	1 (11)	
Unknown	8	190	

These patients differ in terms of prior nephrectomy, bone and liver metastases and number of metastastic site compared to the 578 patients with non-missing IMDC risk score (Supplementary Table 1). Most of patients with missing IMDC risk factors are patients treated at Gustave Roussy after first line treatment. Indeed, more frequently the missing values for IMDC score (detailed in Supplementary Table 1) were biochemical factors. Abbreviations: mRCC, metastatic renal cell carcinoma; DTT, Diagnosis < 1 year; UNL, upper normal level; LNL, low normal level.
